# Prevalence and outcomes of central line-associated bloodstream infections in intensive care units in Saudi Arabia: a systematic review and meta-analysis

**DOI:** 10.3389/fmicb.2026.1854248

**Published:** 2026-06-11

**Authors:** Jehad A. Aldali, Abdullah A. Alshehr, Maysoon A. Abdelhamid, Lana G. Alghamdi, Hanin M. Alhuwaymani, Lamia S. Alharbi, Dhai A. Alazwari, Lojain S. Alkahtani, Wael Y. Khawagi

**Affiliations:** 1 Department of Pathology, College of Medicine, Imam Mohammad Ibn Saud Islamic University (IMSIU), Riyadh, Saudi Arabia; 2 Department of Clinical Pharmacy, College of Pharmacy, Taif University, Taif, Saudi Arabia; 3 College of Medicine, Imam Mohammad Ibn Saud Islamic University (IMSIU), Riyadh, Saudi Arabia

**Keywords:** central-line associated blood stream infection, clinical outcomes, intensive care unit, prevalence, Saudi Arabia

## Abstract

**Background:**

Central line-associated bloodstream infections (CLABSIs) are among the most serious healthcare-associated infections in intensive care units (ICUs), contributing to increased morbidity, mortality, and healthcare burden. However, evidence regarding the epidemiology and outcomes of CLABSI in Saudi Arabian ICUs remains limited. This systematic review and meta-analysis aimed to evaluate the incidence, microbiology, and clinical outcomes of CLABSI among ICU patients in Saudi Arabia.

**Methods:**

A systematic review and meta-analysis were conducted in accordance with the PRISMA guidelines. PubMed, Medline, Embase, and Web of Science were searched for studies published between January 2015 and November 2025. Studies conducted in Saudi Arabian ICUs reporting CLABSI incidence, microbiology, or clinical outcomes were included. Pooled incidence rates were estimated using random-effects meta-analysis. The review protocol was prospectively registered in PROSPERO (CRD420261337969).

**Results:**

Of 2,788 identified records, 11 studies were included in the systematic review and 7 in the meta-analysis. Included studies primarily consisted of retrospective observational, surveillance, and cohort studies conducted across different ICU settings in Saudi Arabia. The pooled CLABSI incidence was 3.05 per 1,000 central line-days (95% CI: 1.85–4.26; *I*^2^ = 96%). Subgroup analyses demonstrated variability according to ICU type, geographic region, and study design. The highest pooled incidence was observed in mixed ICU settings at 3.41 per 1,000 central line-days (95% CI: 1.12–5.69), while individual study estimates ranged from 0.64 to 4.61 per 1,000 central line-days. Studies excluded from the meta-analysis reported CLABSI proportions ranging from 15 to 28.3% of healthcare-associated or ICU-acquired infections. *Klebsiella pneumoniae* and *Acinetobacter baumannii* were the predominant pathogens, with several studies reporting multidrug-resistant isolates. CLABSI was associated with increased mortality, prolonged hospitalization, and greater healthcare burden. High heterogeneity and variability in surveillance methods across studies should be considered when interpreting the pooled estimates.

**Conclusion:**

CLABSI remains a significant healthcare-associated infection among ICU patients in Saudi Arabia and is associated with considerable clinical burden. Variability in incidence across ICU settings highlights the importance of strengthening infection prevention strategies and surveillance systems. Further multicenter studies are needed to improve understanding of risk factors, microbiological patterns, and preventive interventions.

## 1 Introduction

The Center for Disease Control and prevention (CDC) defined Central line-associated bloodstream infection (CLABSI) as a laboratory-confirmed bloodstream infection that develops at least 2 full days after insertion of a central venous catheter (Centers for Disease Control and Prevention, 2022). CLABSI is among the most serious healthcare-associated infections and represents a major challenge in intensive care units (ICUs) because of its association with increased morbidity, mortality, prolonged hospitalization, and healthcare costs (Haddadin et al., 2017).

Previous studies have demonstrated that CLABSI significantly increases the risk of mortality among critically ill patients. Infected patients may have a mortality risk approximately 3.89 times higher than uninfected patients (95% CI: 1.33–11.31; *p* = 0.013) (Mosquera et al., 2024). The presence of antimicrobial-resistant microorganisms may further increase mortality risk, with resistant infections associated with approximately 4-fold higher mortality compared with susceptible organisms (95% CI: 1.17–13.96; *p* = 0.027) (Mosquera et al., 2024). In addition, CLABSI has been associated with prolonged ICU and hospital stay, increased catheter duration, and greater healthcare expenditure (Kaye et al., 2014).

CLABSI can be caused by different pathogens of microorganisms such as Gram negative bacteria, and Gram positive bacteria (Fagan et al., 2013). A prospective study conducted in a specialized hospital in New Delhi reported that *Klebsiella pneumoniae* was the most common pathogen, followed by coagulase-negative staphylococci, *Staphylococcus aureus*, and *Acinetobacter baumannii* (Arunan et al., 2023).

The study also reported a high prevalence of multidrug-resistant organisms and biofilm-producing isolates, highlighting the growing challenge of antimicrobial resistance in ICU settings (Arunan et al., 2023). Critically ill patients in the Intensive Care Unit (ICU) frequently require prolonged central venous catheterization for hemodynamic monitoring, medication administration, and supportive care. Several factors contribute to increased CLABSI risk in these patients, including prolonged catheter duration, severity of illness, invasive procedures, and increased device dependence (Stewart et al., 2023). A study conducted in a university hospital ICU in Japan identified longer ICU stays, prolonged catheterization duration, and higher APACHE II scores as independent predictors of CLABSI (Moriyama et al., 2022).

International surveillance studies have demonstrated substantial variation in CLABSI incidence across healthcare systems and regions. Multinational data from ICUs across nine countries reported a pooled CLABSI incidence of 5.08 per 1,000 catheter-days (Rosenthal et al., 2024), whereas substantially lower rates have been reported in healthcare systems with mature infection prevention programs, such as the United States (Toor et al., 2022). Meanwhile, data from the Victorian Healthcare Association in Australia showed that 581 newborns contracted either central line or peripheral line associated bloodstream infections, corresponding to an average peripheral catheter-associated bloodstream infection incidence of 0.60 per 1,000 peripheral catheter days and an average CLABSI rate of 2.26 per 1,000 central catheter days (Liu et al., 2025). These differences may reflect better prevention bundles and health care surveillance in the U.S. (Rosenthal et al., 2024; Toor et al., 2022; Liu et al., 2025).

Within Gulf Cooperation Council (GCC) countries, CLABSI incidence rates remain higher than those reported by the National Healthcare Safety Network (NHSN), although lower than rates reported by the International Nosocomial Infection Control Consortium (INICC) (Balkhy et al., 2017). A national surveillance program conducted in Saudi Arabia reported 1,542 CLABSI events over 2 years, corresponding to an overall incidence rate of 3.24 per 1,000 central line-days. Although Saudi hospitals demonstrated rates comparable to GCC benchmarks, substantial variability across institutions and ICU settings was observed (Alanazi et al., 2021). Despite the growing burden of healthcare-associated infections, comprehensive evidence regarding the epidemiology, microbiology, and clinical outcomes of CLABSI in Saudi Arabian ICUs remains limited (AlSaleh et al., 2023). Therefore, this systematic review and meta-analysis aimed to synthesize the available evidence on CLABSI incidence, causative organisms, and associated clinical outcomes among ICU patients in Saudi Arabia.

## 2 Methods

### 2.1 Protocol and guidance

The study protocol was conducted in accordance with the PRISMA and Cochrane Collaboration Handbook guidelines (Page et al., 2021; Higgins et al., 2019) and was registered in PROSPERO (CRD420261337969).

### 2.2 Search strategies

A comprehensive systematic search was conducted in PubMed, MEDLINE, Embase, and Web of Science for studies published between January 2015 and November 2025. Search strategies combined controlled vocabulary (e.g., MeSH terms) and free-text keywords related to CLABSI, central venous catheters, intensive care units, and Saudi Arabia using Boolean operators and database-specific syntax. The reference lists of included studies were additionally screened for potentially relevant publications. Full database-specific search strategies are provided in Supplementary Table S1.

### 2.3 Inclusion criteria

To be eligible for full-text review, studies had to be conducted in Saudi Arabia, include patients admitted to intensive care units (ICUs) with central venous catheters, and report data related to CLABSI prevalence, incidence, outcomes, catheter characteristics, epidemiology, or microbiology. Eligible study designs included randomized controlled trials (RCTs), observational studies, cohort studies, controlled clinical trials, cross-sectional studies, retrospective studies, and prospective studies.

### 2.4 Exclusion criteria

Studies exclusively focused on fungal or viral bloodstream infections, non-bloodstream infections, or non-ICU settings were excluded. However, studies reporting fungal pathogens as part of overall CLABSI microbiology were eligible for inclusion. Additional exclusions included animal studies, studies conducted outside Saudi Arabia, non-English publications, review articles, case reports, and case series.

### 2.5 Study selection and data extraction

The systematic review screening was conducted using the search results that were exported to Rayyan QCRI. The titles and abstracts were screened by four groups of two examiners, and duplicates were removed. A third reviewer resolved conflicts. Data from the eligible studies were independently extracted into a standardized Excel document, which summarized study details such as the authors, publication year, design, location, setting, sample criteria, size, demographics, interventions, and outcomes.

### 2.6 Risk of bias assessment

The risk of bias was evaluated using critical appraisal instruments specific to each study design developed by the Joanna Briggs Institute (JBI) critical appraisal checklist according to each study design (Jordan et al., 2019). The assessment included domains related to participant selection, measurement of outcomes, identification and control of confounding factors, completeness of follow-up, and appropriateness of statistical analysis. Two reviewers independently assessed each study, and disagreements were resolved through discussion or consultation with a third reviewer. A detailed domain-level risk-of-bias assessment for each included study is presented in Supplementary Table S2. Sensitivity analysis excluding studies with a high risk of bias was conducted to evaluate the robustness of the pooled estimates.

### 2.7 Statistical analysis

Given the hierarchical structure of the data, a multilevel meta-analysis was conducted to account for the dependence among multiple effect sizes derived from the same studies (Houben et al., 2015; Spruit et al., 2020). Several included studies contributed multiple CLABSI incidence estimates across different ICU settings or subgroups, resulting in statistically dependent effect sizes within the same study (Van den Noortgate et al., 2013). A conventional random-effects meta-analysis assumes independence of effect sizes and was therefore considered insufficient for the hierarchical structure of the dataset.

In this framework, Level 1 represented the sampling variance, Level 2 captured within-study variability among multiple effect sizes from the same study and Level 3 accounted for between-study heterogeneity. Variance components were estimated using restricted maximum likelihood (REML) (Cheung, 2014).

All statistical analyses were conducted using R statistical software (version 4.4.1) with the meta package, specifically the metarate function. To preserve the clinical interpretability of infection-control benchmarks, incidence rates were pooled directly on the raw, untransformed scale (sm = “IR”) using inverse-variance weighting. CLABSI incidence was expressed as cases per 1,000 central line-days. No additional transformation methods were applied because no zero-event studies were identified. Pooled CLABSI incidence rates and corresponding 95% confidence intervals (95% CIs) were estimated, and between-study heterogeneity was assessed using the *I*^2^ statistic. Subgroup analyses were conducted according to ICU type, geographic region, and study design. Formal statistical comparisons between subgroups were not performed because of differences in sample size and reporting methods across studies.

A leave-one-out sensitivity analysis was performed to assess the robustness of the pooled CLABSI incidence estimates. Given the hierarchical structure of the dataset, with multiple ICU types nested within individual studies, the sensitivity analysis was conducted at the study level to evaluate whether any single study had a disproportionate influence on the pooled estimate and to identify potential outliers (Mao et al., 2025). The leave-one-out analysis demonstrated stable pooled incidence estimates, with all recalculated values remaining within the 95% confidence interval of the primary model.

## 3 Results

### 3.1 Study selection

**Figure 1:**
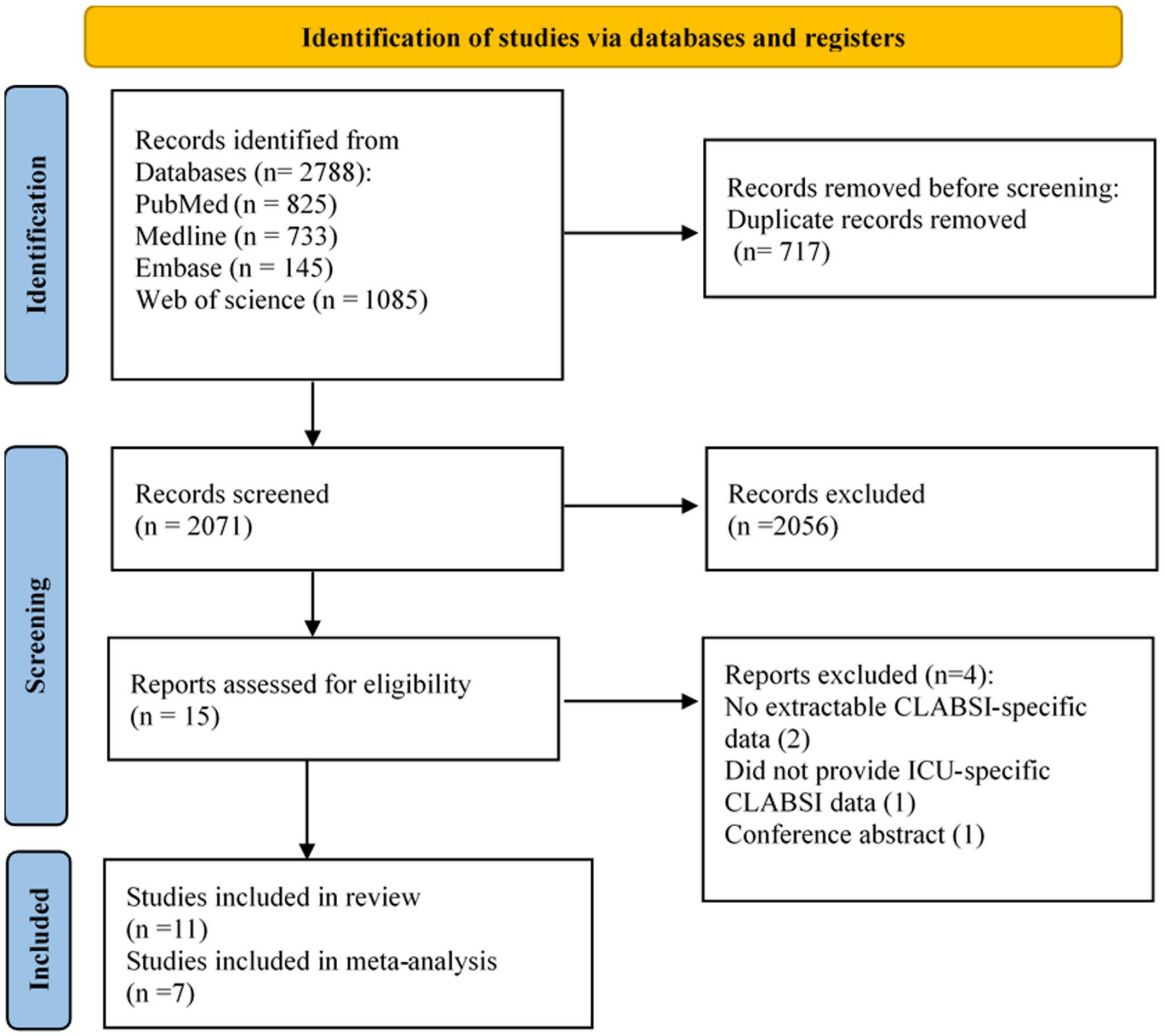
PRISMA flow diagram of the study selection process.

The systematic search identified 2,788 potentially relevant studies published between 2015 and 2025. After removing duplicates, titles and abstracts were screened, followed by full-text assessment against predefined eligibility criteria. In the end, 11 studies met the criteria for inclusion and were included in the systematic review. Of these, seven were eligible for inclusion in the meta-analysis. The study selection process is summarized in the PRISMA flow diagram (Figure 1).

### 3.2 Characteristics of included studies

The included studies were conducted across multiple regions in Saudi Arabia, including Riyadh (*n* = 3) (Albudayri et al., 2024; Alshamrani et al., 2024; Elabbasy et al., 2024), Jeddah (*n* = 2) (Al-Sofyani and Uddin, 2022; Almazeedi et al., 2023), Taif (*n* = 1) (Mazi et al., 2021), Al-Ahsa (*n* = 1) (AlSaleh et al., 2023), Al-Khobar (*n* = 1) (Alwazzeh et al., 2023), Dhahran (*n* = 1) (AlSaleh et al., 2023; Al-Tawfiq et al., 2023), Hail (*n* = 1) (Saleem et al., 2023), and Aljouf (*n* = 1) (Alshammari and Alruwaili, 2025). Most studies were single-center (*n* = 10), with only one multicentre study conducted across multiple hospitals in the Al-Ahsa region (AlSaleh et al., 2023). In terms of study design, the majority were retrospective observational or surveillance studies (*n* = 7) (AlSaleh et al., 2023; Albudayri et al., 2024; Al-Sofyani and Uddin, 2022; Almazeedi et al., 2023; Alwazzeh et al., 2023), while fewer studies employed prospective designs (*n* = 2) (AlSaleh et al., 2023; Mazi et al., 2021; Alwazzeh et al., 2023; Saleem et al., 2023) or cross-sectional approaches (*n* = 2) (Alshamrani et al., 2024; Alshammari and Alruwaili, 2025). Study populations varied across ICU settings, including mixed ICUs (*n* = 6) (AlSaleh et al., 2023; Alshamrani et al., 2024; Mazi et al., 2021; Alshammari and Alruwaili, 2025), adult ICUs (*n* = 2) (Albudayri et al., 2024; Saleem et al., 2023), pediatric ICUs (*n* = 2) (Al-Sofyani and Uddin, 2022; Almazeedi et al., 2023), and neonatal ICUs (*n* = 1) (Elabbasy et al., 2024).

Several studies applied standardized CDC/NHSN surveillance definitions for CLABSI diagnosis, whereas others either did not clearly report diagnostic criteria or used alternative definitions such as catheter-related bloodstream infection (CRBSI). This variability in diagnostic and surveillance definitions may have contributed to the substantial clinical and methodological heterogeneity observed across studies.

Reporting formats also varied, with outcomes expressed as incidence density per 1,000 central line-days (*n* = 5) (AlSaleh et al., 2023; Alshamrani et al., 2024; Al-Sofyani and Uddin, 2022; Mazi et al., 2021; Al-Tawfiq et al., 2023), total number of events (*n* = 4) (Almazeedi et al., 2023; Alwazzeh et al., 2023; Saleem et al., 2023), or proportions of infected patients without standardized denominators (*n* = 2) (Elabbasy et al., 2024; Alshammari and Alruwaili, 2025) (Table 1).

**Table 1:** Characteristics of included studies reporting central line-associated bloodstream infections.

Author	Region	Study design	Study period	Study setting	ICU population	CLABSI diagnostic criteria/definition	Patients with Central line (n)	CLABSI events	Central-line days (Denominator)	Age in years (mean ± SD, mean (range) or median [IQR])	Sex (Female %)	Eligible for meta-analysis
Alshammari and Alruwaili (2025)	Aljouf	Retrospective cross-sectional	Jan 2020–Dec 2023	Single center	Mixed ICU	CDC/NHSN definition	208	7	NR	58 (1–118)	43.5	No
Elabbasy et al. (2024)	Riyadh	Prospective cross-sectional	Jul 2022–Jul 2023	Single center	Neonatal ICU	NR	415	26	5,640	NR	53.5	Yes
Albudayri et al. (2024)	Riyadh	Retrospective cross-sectional	Jan 2022–Feb 2024	Single center	Adult ICU	NR	NR	21	NR	62.9 ± 15.1	61.9	No
AlSaleh et al. (2023)	Al-Ahsa	Retrospective surveillance	Jan–Dec 2022	Multicenter	Mixed ICU	CDC/NHSN definition	NR	56	13,042	NR	NR	Yes
Alwazzeh et al. (2023)	Al-Khobar	Retrospective observational	Jan 2015–Dec 2020	Single center	Mixed ICU	CDC/NHSN definition	NR	125	32,560	Adults: 58 (17–95) Children: 7 (2–168) months Neonates: 17 (10–30) days	41.6	Yes
Almazeedi et al. (2023)	Jeddah	Retrospective cross-sectional	2021–2022	Single center	Pediatric ICU	CDC/NHSN definition	NR	41	NR	3.5 ± 4.2	NR	No
Al-Sofyani and Uddin (2022)	Jeddah	Retrospective cohort	Jan 2017–Jan 2018	Single center	Pediatric ICU	CRBSI definition based on differential time to positivity (DTP)	145	6	1,463	12 [4–72] months	42.8	Yes
Mazi et al. (2021)	Taif	Prospective cohort	Jan 2017–Dec 2019	Single center	Mixed ICU	CDC/NHSN definition	NR	15	23,595	51 ± 26.5	NR	Yes
Al-Tawfiq et al. (2023)	Dhahran	Retrospective surveillance	Jan 2017–Dec 2020	Single center	Mixed ICU	CDC/NHSN definition	NR	30	9,847	NR	NR	Yes
Alshamrani et al. (2024)	Riyadh	Retrospective surveillance	Jan 2018–Dec 2021	Single center	Mixed ICU	CDC/NHSN definition	NR	156	46,406	50.4 ± 28.5	51.9	Yes
Saleem et al. (2023)	Hail	Prospective observational	Jan–Dec 2019	Single center	Adult ICU	NR	NR	25	NR	NR	NR	No

CLABSI, central line-associated bloodstream infection; ICU, intensive care unit; CDC, Centers for Disease Control and Prevention; NHSN, National Healthcare Safety Network; CRBSI, catheter-related bloodstream infection; NR, not reported.

### 3.3 The CLABSI rates across ICUs

The incidence of CLABSI varied substantially across studies and ICU settings. When reported as incidence density, rates ranged from 0.64 to 4.61 per 1,000 central line-days (Alshamrani et al., 2024; Al-Sofyani and Uddin, 2022; Mazi et al., 2021; Al-Tawfiq et al., 2023). Some studies reported only the number of CLABSI events without standardization to central line-days (Albudayri et al., 2024; Almazeedi et al., 2023; Saleem et al., 2023; Alshammari and Alruwaili, 2025), therefore, these studies were not included in the meta-analysis. CLABSI was reported as a proportion of healthcare-associated or ICU-acquired infections (15–28.3%) across studies excluded from the meta-analysis, whereas one study reported only case counts (*n* = 21) without a denominator.

**Figure 2:**
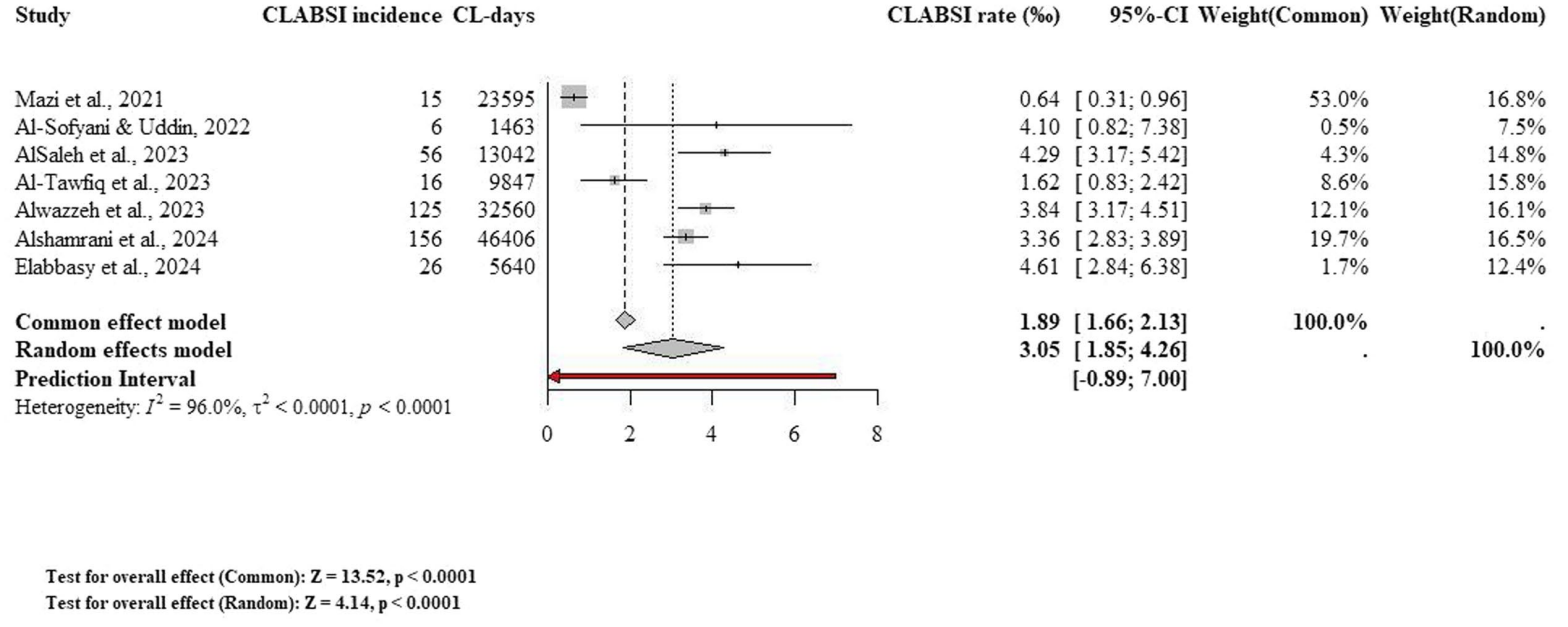
The pooled and weighted CLABSI rates across ICUs.

The pooled CLABSI incidence rate in Saudi Arabian ICUs, derived from seven included studies, was 3.05 per 1,000 CL-days (95% CI: 1.85–4.26; *p* < 0.0001). The analysis showed significant heterogeneity (*Q* = 149.28, df = 6, *p* < 0.0001), with an *I*^2^ of 96.0%. Variance partitioning indicated that the majority of this variability was attributed to between-study differences (*τ*^2^ < 0.0001). Variability across studies was observed in ICU populations, study designs, surveillance methodologies, outcome reporting formats, and CLABSI diagnostic definitions. Figure 2 depicts the pooled and weighted CLABSI rates across ICUs.

### 3.4 The CLABSI rates in different types of ICUs

**Figure 3:**
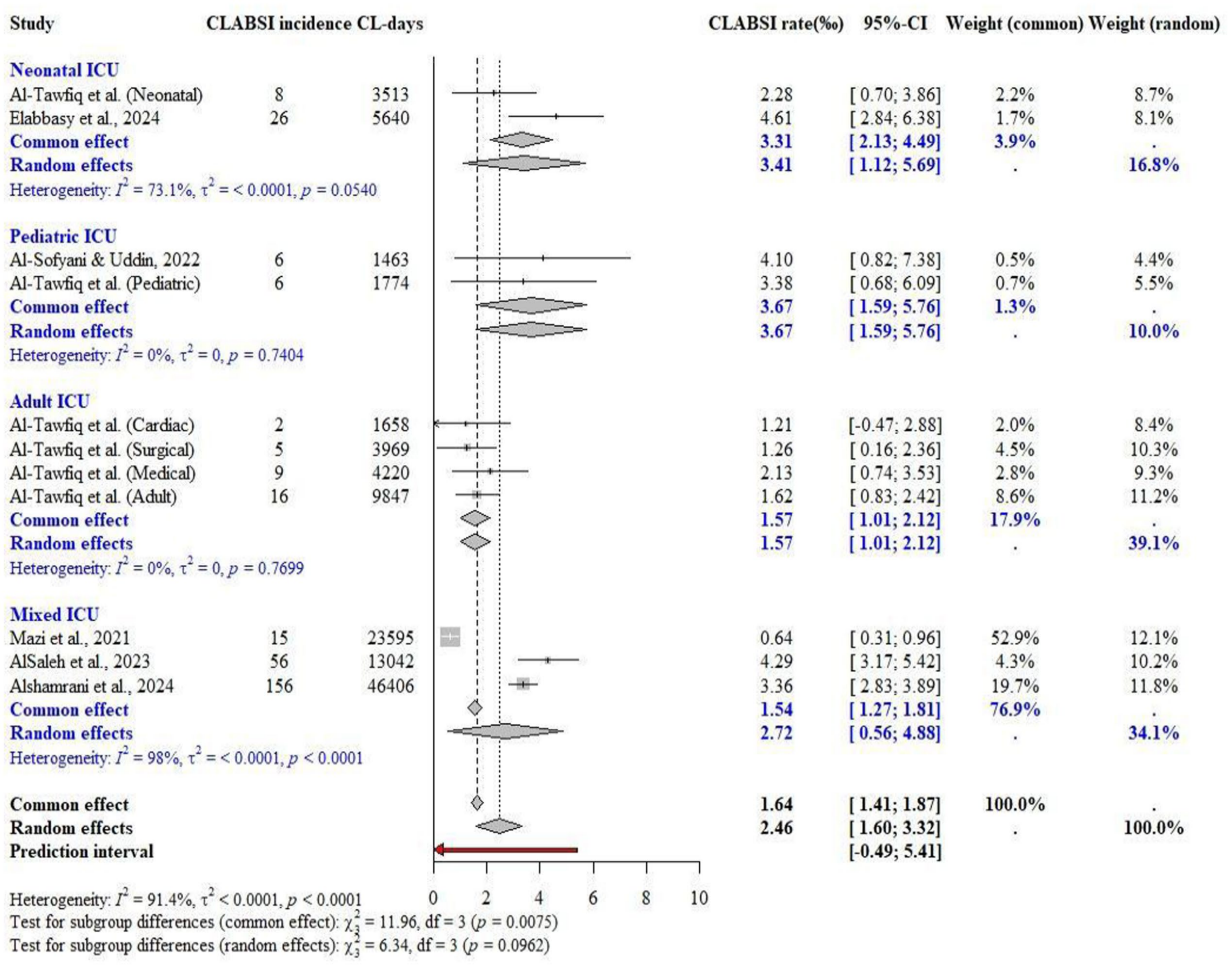
The pooled and weighted CLABSI rates in different types of ICUs.

Clinical trends differed amongst ICU types, according to subgroup analysis. The Neonatal ICU had the highest pooled CLABSI rate at 3.41 per 1,000 CL-days (95% CI: 1.12–5.69), followed by the Pediatric ICU at 3.67 per 1,000 CL-days (95% CI: 1.59–5.76). The Mixed ICU group had a rate of 2.72 per 1,000 CL-days (95% CI: 0.56–4.88‰), whereas the Adult ICU had the lowest pooled rate of 1.57 per 1,000 CL-days (95% CI: 1.01–2.12‰). The test for subgroup differences using the random effects model revealed *χ*^2^ = 6.34, df = 3, *p* = 0.0962. While the common effect model revealed considerable variation (*p* = 0.0075), the random effects model shows that the variations between ICU types do not meet the conventional threshold for statistical significance. Figure 3 depicts the pooled and weighted CLABSI rates for the different ICU classifications.

### 3.5 The CLABSI rates in ICUs of different geographical regions

**Figure 4:**
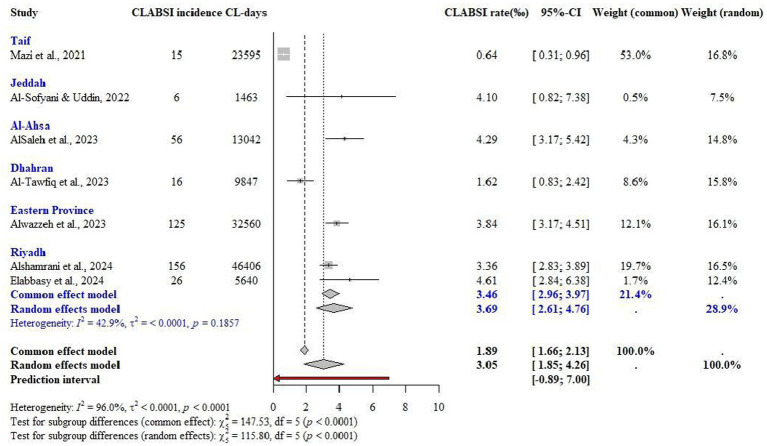
The pooled and weighted CLABSI rates in different regions of the Kingdom of Saudi Arabia.

According to the random effects model, the Al-Ahsa region recorded the highest individual study rate at 4.29 per 1,000 CL-days (95% CI: 3.17–5.42‰), followed closely by Riyadh, which showed a pooled regional rate of 3.46 per 1,000 CL-days (95% CI: 2.96–3.97‰). In contrast, the lowest incidence was observed in the Taif region, with a rate of 0.64 per 1,000 CL-days (95% CI: 0.31–0.96). Significant heterogeneity was identified across regional subgroup analyses (*I*^2^ = 96.0%, *p* < 0.0001). Although subgroup analyses suggested variability in CLABSI incidence across regions, these findings should be interpreted cautiously because several subgroups included a limited number of studies. Figure 4 provides a detailed description of pooled and weighted CLABSI rates for different regions of the Kingdom of Saudi Arabia.

### 3.6 The CLABSI rates in different types of study design

**Figure 5:**
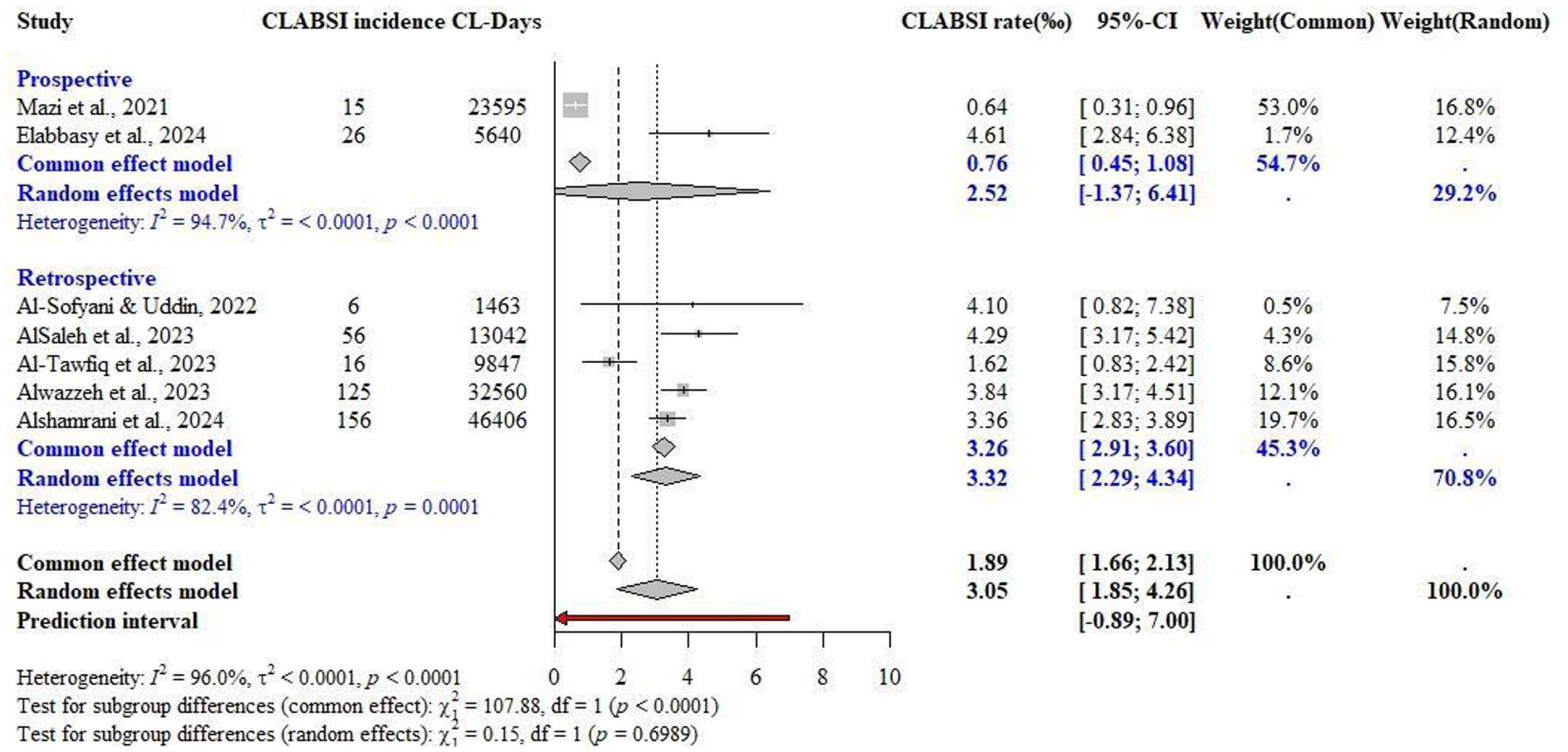
The pooled and weighted CLABSI rates in different types of study design.

Subgroup analysis according to research design revealed clear differences. A pooled rate of 2.52 (95% CI: 0.00–6.41) was found in prospective trials; however, this subgroup showed very high heterogeneity (*I*^2^ = 94.7%) and a broad confidence range. Retrospective studies, on the other hand, indicated a pooled incidence of 3.32 (95% CI: 2.29–4.34), which was higher and more consistent. The statistical test for subgroup differences using the random effects model revealed no significant difference between prospective and retrospective designs (*χ*^2^ = 0.15, df = 1, *p* = 0.6989), despite the apparent difference in rates. Figure 5 provides a detailed breakdown of these pooled rates and associated heterogeneity by study design.

### 3.7 Clinical outcomes

Mortality outcomes were reported in four studies (Albudayri et al., 2024; Alshamrani et al., 2024; Almazeedi et al., 2023; Alwazzeh et al., 2023). Overall mortality rates varied substantially, ranging from 29.4 to 76.2%. CLABSI-attributable mortality was reported in two studies, with estimates of 14.0 and 48.8% (Almazeedi et al., 2023; Alwazzeh et al., 2023).

### 3.8 Length of stay

Length of stay was reported in four studies (Alshamrani et al., 2024; Al-Sofyani and Uddin, 2022; Saleem et al., 2023), with substantial variability in reporting formats and outcomes. One study found that the median length of stay in the ICU was 43 days (IQR 25.8–79.3). Patients with peripherally inserted central catheters stayed in the ICU for much longer than those with umbilical venous catheters (54 vs. 34 days, *p* < 0.001) (Moriyama et al., 2022). A different study found that the average stay in the ICU was 45.2 ± 86.3 days and the average stay in the hospital was 95.5 ± 171.0 day (Alshamrani et al., 2024). Categorical reporting was also observed. One study indicated that 76.6% of patients had a length of stay exceeding 10 days, while 71% had a central line duration of less than 2 weeks (Al-Sofyani and Uddin, 2022). Another study reported that the majority of patients experienced prolonged stays of >2 weeks (69.7%), compared to 30.3% with stays of 1–2 weeks (*p* = 0.025, 0.004) (Saleem et al., 2023).

### 3.9 Time to CLABSI onset

Time to CLABSI onset was reported in two studies (Elabbasy et al., 2024; Al-Sofyani and Uddin, 2022). One study reported a mean time to onset of 14 ± 7.6 days (range 7–26), while another described variation in pre-CLABSI duration across patient groups, with median durations of 21 days in adults, 32 days in children, and 15 days in neonates.

### 3.10 Microbiological characteristics

Microbiological data were reported in seven studies (Albudayri et al., 2024; Alshamrani et al., 2024; Almazeedi et al., 2023; Alwazzeh et al., 2023; Saleem et al., 2023; Alshammari and Alruwaili, 2025). Gram-negative organisms predominated across most studies, accounting for 80–100% of isolates in several reports. In contrast, Gram-positive organisms were less frequently reported, ranging from 0% to approximately 33%. *Klebsiella pneumoniae* was consistently identified as the most common causative organism. Other frequently reported pathogens included *Acinetobacter baumannii* and *Pseudomonas aeruginosa*. Antimicrobial resistance data were reported in three studies (Albudayri et al., 2024; Almazeedi et al., 2023; Alwazzeh et al., 2023). The prevalence of multidrug-resistant (MDR) organisms ranged from 25.5 to 45%.

### 3.11 Risk factors and clinical characteristics

Date on comorbidities were reported in seven of the included studies (Albudayri et al., 2024; Elabbasy et al., 2024; Almazeedi et al., 2023; Alwazzeh et al., 2023; Saleem et al., 2023; Alshammari and Alruwaili, 2025). A high burden of comorbid conditions was consistently observed, with 82–85% of patients having at least one comorbidity (Alshamrani et al., 2024; Alshammari and Alruwaili, 2025). One study described a distribution in which 31.4% of patients had ≥3 comorbidities. Another study reported that having ≥3 comorbidities was significantly associated with increased mortality (*p* < 0.05) (Alwazzeh et al., 2023). Common comorbidities included hypertension (30–66.7%) and diabetes mellitus (30–71.4%), both of which were frequently reported across studies. In one pediatric study, low birth weight was the most prevalent condition (59.8%), followed by gastrointestinal disorders (14%) (Elabbasy et al., 2024).

### 3.12 Risk of bias

The methodological quality of the included studies was assessed using the JBI critical appraisal tools appropriate for each study design. Overall, most included studies demonstrated low-to-moderate risk of bias. Common methodological limitations encompassed insufficient adjustment for confounding variables, restricted application of multivariable analyses, and incomplete disclosure of exposure measurement techniques. Two studies were judged to have a high risk of bias and were not eligible for inclusion in the meta-analysis due to insufficient data for quantitative synthesis. A detailed domain-level risk-of-bias assessment for each included study is presented in Supplementary Table S2.

## 4 Discussion

This systematic review and meta-analysis provide a comprehensive synthesis of CLABSI rates across intensive care units in Saudi Arabia. The findings demonstrate that CLABSI remains a clinically significant and variable healthcare-associated infection across ICU settings in the country. The pooled incidence rate of 3.05 per 1,000 central line-days highlights a considerable burden, accompanied by substantial heterogeneity across studies. In addition to variability in incidence, the included studies consistently report significant clinical consequences, including high mortality rates, prolonged hospital stays, and frequent involvement of multidrug-resistant organisms.

The pooled CLABSI rate identified in this review is broadly comparable to rates reported internationally but remains higher than those observed in healthcare systems with mature infection prevention programs. For example, data from the United States National Healthcare Safety Network (NHSN) have shown substantially lower CLABSI rates recently, largely due to widespread implementation of prevention bundles and surveillance systems (Al-Tawfiq et al., 2023). Similarly, international benchmarking studies have demonstrated that standardized infection control interventions can reduce CLABSI rates to less than 1.0 per 1,000 catheter-days in high-performing units (Fakih et al., 2017).

The substantial heterogeneity observed in this review (*I*^2^ = 96%) indicates considerable variability across the included studies and limits the interpretability of the pooled CLABSI incidence estimate. Therefore, the pooled findings should be considered exploratory rather than definitive. Several factors likely contributed to the heterogeneity, including differences in ICU populations (adult, pediatric, neonatal, and mixed ICUs), study designs, surveillance methodologies, diagnostic criteria, denominator reporting, and outcome definitions. In addition, variability in surveillance quality and reporting practices across institutions may further reduce comparability between studies. Although several studies applied CDC/NHSN surveillance definitions, others relied on CRBSI-based criteria or did not clearly describe diagnostic methods, which may have influenced the reported incidence rates (Rai et al., 2023).

Subgroup analysis suggested higher CLABSI rates in neonatal and pediatric ICUs compared with adult ICUs, although these differences were not statistically significant. This trend aligns with existing literature, which identifies neonates and pediatric patients as high-risk populations due to immature immune systems, low birth weight, and the technical challenges of central line management (McCourt, 1994). Prolonged catheter use and increased device dependence in these populations further contribute to infection risk. Interpretation of subgroup analyses should also be approached cautiously because several subgroup categories were based on a limited number of studies, which may reduce the reliability of subgroup-specific estimates.

The significant regional variation observed in this review is also consistent with international evidence, which shows that CLABSI rates vary widely across institutions and geographic regions depending on healthcare resources, staffing, infection control practices, and surveillance systems (Rosenthal et al., 2010). Differences in reporting practices and diagnostic rigor may also contribute to this variability. Interpret these findings within the context of the Saudi healthcare system and ongoing national infection prevention initiatives. Recently, Saudi Arabia has expanded ICU capacity and strengthened healthcare quality programs through national healthcare transformation efforts. National surveillance systems and infection prevention programs have enhanced monitoring of healthcare-associated infections, including CLABSI, across hospitals and ICUs. In addition, several healthcare institutions participate in international benchmarking systems such as NHSN and INICC. Nevertheless, variability in surveillance practices, reporting standards, and ICU resources across institutions may contribute to differences in reported CLABSI rates (Hodge, 2024).

The predominance of Gram-negative organisms in this review is particularly noteworthy. While Gram-positive organisms have historically been the leading cause of CLABSI in many high-income settings, recent studies from the Middle East and other regions have reported an increasing predominance of Gram-negative pathogens, including *Klebsiella pneumoniae*, *Acinetobacter baumannii*, and *Pseudomonas aeruginosa* (Manandhar, 2022). This shift is often associated with higher rates of antimicrobial resistance and presents additional challenges for clinical management. The reported prevalence of multidrug-resistant organisms in this review (25.5–45%) is consistent with global concerns regarding the growing burden of antimicrobial resistance in ICU settings (Marino et al., 2025).

The literature also strongly supports the clinical impact of CLABSI, as observed in this review. Previous studies have demonstrated that CLABSI is associated with increased mortality, prolonged ICU and hospital stays, and higher healthcare costs (Chovanec et al., 2021). Attributable mortality rates vary widely but can be substantial, particularly in critically ill populations. Additionally, longer catheter duration has been consistently identified as a key risk factor for infection, supporting the findings on time to CLABSI onset in this review (Lafuente Cabrero et al., 2023).

The findings of this review reinforce the importance of implementing evidence-based CLABSI prevention strategies. Central line insertion and maintenance bundles, including hand hygiene, maximal sterile barrier precautions, chlorhexidine skin antisepsis, and daily review of line necessity, have been shown to significantly reduce infection rates when consistently applied (Awad, 2024). In addition, standardization of surveillance using CDC/NHSN definitions is essential to improve comparability and benchmarking across institutions. National and institutional surveillance programs, combined with regular feedback and auditing, have been associated with sustained reductions in CLABSI rates (McKibben et al., 2005).

The predominance of Gram-negative and multidrug-resistant organisms emphasizes the necessity to integrate infection prevention with antimicrobial stewardship programs. Appropriate empirical therapy guided by local antibiograms and efforts to minimize unnecessary antibiotic use are critical components of effective CLABSI management (Toma et al., 2025). Targeted prevention strategies for high-risk populations, such as patients with multiple comorbidities, prolonged ICU stays, or extended catheter use, may further enhance outcomes.

Future studies should prioritize multicentre prospective designs using standardized CDC/NHSN definitions and consistent reporting of incidences per 1,000 central line-days. Research is also needed to identify independent risk factors for CLABSI using adjusted analyses, as well as to evaluate the effectiveness of prevention interventions in the Saudi healthcare context. Interventional studies assessing bundle compliance, staff education, and antimicrobial stewardship programs would be particularly valuable.

Ongoing surveillance of microbiological patterns and antimicrobial resistance trends is also critical, given the predominance of Gram-negative organisms identified in this review. Additionally, future studies should report patient-centerd outcomes, including attributable mortality, length of stay, and economic burden, using standardized measures.

This review provides a comprehensive synthesis of CLABSI epidemiology across multiple ICU settings in Saudi Arabia. The inclusion of meta-analysis and subgroup analyses enhances the robustness of the findings, while the integration of clinical and microbiological data provides a holistic perspective. However, the findings should be interpreted in light of several limitations. The small number of included studies and substantial heterogeneity limit the precision of pooled estimates. Publication bias assessment was not performed because the small number of studies included in the meta-analysis limits the reliability and interpretability of funnel plots and Egger’s regression testing. Variability in definitions and reporting methods further reduces comparability. Most studies were single-center and retrospective, limiting generalizability and increasing the risk of bias. Inadequate adjustment for confounding variables was also a common limitation. Despite these challenges, the review highlights important gaps in the literature and provides a foundation for future research and quality improvement initiatives aimed at reducing CLABSI’s burden in Saudi Arabia.

## 5 Conclusion

CLABSI remains an important healthcare-associated infection among ICU patients in Saudi Arabia and is associated with substantial clinical burden, including increased mortality and prolonged hospitalization. The included studies demonstrated variability in reported incidence rates, microbiological patterns, and clinical outcomes across ICU settings. However, interpretation of the pooled findings should be approached cautiously because of the high heterogeneity and methodological variability among included studies. Further multicenter studies using standardized surveillance definitions and reporting methods are needed to better characterize the epidemiology of CLABSI and support infection prevention efforts in Saudi ICUs.

## Data Availability

The raw data supporting the conclusions of this article will be made available by the authors, without undue reservation.
