# Supplementary material for: Prevalence and outcomes of central line-associated bloodstream infections in intensive care units in Saudi Arabia: a systematic review and meta-analysis
Source: Front Microbiol. 2026 Jun 11;17:1854248. doi: 10.3389/fmicb.2026.1854248 (PMC13294151; doi:10.3389/fmicb.2026.1854248)
Supplement: Supplementary file 1 [file Table_1.DOCX]

**Supplementary Table 1 &2**

**Supplementary Table S1. Complete Search Strategies Used for Each Database**

| **DATABASES** | **SEARCH STRATEGY** | **IDENTIFIED STUDIES**  **(N)** |
| --- | --- | --- |
| **PubMed (NCBI)** | ("Catheter-Related Infections"[MeSH] OR "Central Venous Catheters"[MeSH] OR "Catheterization, Central Venous"[MeSH] OR "Bacteremia"[MeSH] OR "Sepsis"[MeSH] OR "Cross Infection"[MeSH] OR "central line-associated bloodstream infect*"[tiab] OR "catheter-related bloodstream infect*"[tiab] OR CLABSI[tiab] OR CRBSI[tiab] OR "central venous catheter infect*"[tiab] OR "healthcare-associated infect*"[tiab] OR nosocomial[tiab] OR "central venous access"[tiab] OR "peripherally inserted central catheter*"[tiab] OR PICC[tiab] OR "central line"[tiab]) AND ("Intensive Care Units"[MeSH] OR ICU[tiab] OR "intensive care"[tiab] OR "critical care"[tiab]) AND ("Saudi Arabia"[Mesh] OR "Saudi Arabia"[tiab] OR Saudi[tiab] OR KSA[tiab] OR Riyadh[tiab] OR Jeddah[tiab] OR Makkah[tiab] OR Madinah[tiab] OR Dammam[tiab] OR Khobar[tiab] OR Qassim[tiab] OR Hail[tiab] OR Tabuk[tiab] OR Taif[tiab] OR Najran[tiab] OR Abha[tiab] OR "Al-Ahsa"[tiab] OR "Eastern Province"[tiab]) | **825** |
| **MEDLINE** | (central line* or central venous catheter* or CVC or intravascular catheter*).ti,ab.  (bloodstream infection* or bacteremia or septicaemia or septicemia).ti,ab.  (CLABSI or CRBSI or catheter-related infection* or line-associated infection*).ti,ab.  (ICU* or "intensive care" or "critical care").ti,ab.  (Saudi Arabia or KSA or "Kingdom of Saudi Arabia").ti,ab.  (Riyadh or Jeddah or Makkah or Mecca or Madinah or Medina or Dammam or Abha or Taif or Qassim or Al-Qassim or Hail or Najran or Al-Baha or "Al Ahsa" or Ahsa or Khobar or "Al Khobar" or Jubail or Buraidah or Tabuk or Jazan or Jizan or Skaka or Sakaka or Arar or "Northern Borders" or "Eastern Province" or "Western Region" or "Central Region" or "Southern Region").ti,ab. | **733** |
| **EMBASE** | (central line* or central venous catheter* or CVC or intravascular catheter*).ti,ab.  (bloodstream infection* or bacteremia or septicaemia or septicemia).ti,ab.  (CLABSI or CRBSI or catheter-related infection* or line-associated infection*).ti,ab.  (ICU* or "intensive care" or "critical care").ti,ab.  (Saudi Arabia or KSA or "Kingdom of Saudi Arabia").ti,ab.  (Riyadh or Jeddah or Makkah or Mecca or Madinah or Medina or Dammam or Abha or Taif or Qassim or Al-Qassim or Hail or Najran or Al-Baha or "Al Ahsa" or Ahsa or Khobar or "Al Khobar" or Jubail or Buraidah or Tabuk or Jazan or Jizan or Skaka or Sakaka or Arar or "Northern Borders" or "Eastern Province" or "Western Region" or "Central Region" or "Southern Region").ti,ab. | **145** |
| **Science Citation Index Expanded (Web of Science Core Collection) (1900)** | TS=("Central Venous Catheter*" OR CVC OR "central venous access" OR "central line" OR "peripherally inserted central catheter*" OR PICC OR "catheter-related bloodstream infect*" OR "central line-associated bloodstream infect*" OR CLABSI OR CRBSI OR "central venous catheter bloodstream infect*" OR bacteremia OR septicemia OR sepsis OR "healthcare-associated infect*" OR "hospital-acquired infect*" OR "nosocomial infect*") AND TS=("intensive care" OR ICU OR "critical care") AND TS=("Saudi Arabia" OR Saudi OR KSA OR Riyadh OR Jeddah OR Makkah OR Madinah OR Dammam OR Khobar OR Qassim OR Hail OR Tabuk OR Taif OR Najran OR Abha OR "Al-Ahsa" OR "Eastern Province") | **1085** |

**Supplementary Table S2. Domain-level risk-of-bias assessment of included studies using Joanna Briggs Institute (JBI) critical appraisal tools.**

| Study | Study Design | JBI Tool Used | Inclusion Criteria Clearly Defined / Appropriate Sample Frame | Study Subjects and Setting Described | Exposure Measured Validly and Reliably | Standard Criteria Used for Outcome Measurement / Condition Identification | Confounding Factors Identified | Strategies to Address Confounding | Outcomes Measured Validly and Reliably | Follow-Up Adequate and Complete | Appropriate Statistical Analysis | Overall Risk |
| --- | --- | --- | --- | --- | --- | --- | --- | --- | --- | --- | --- | --- |
| Alshammari and Alruwaili, 2025 | Retrospective cross-sectional study | JBI Analytical Cross-Sectional Checklist | Yes | Yes | Unclear | Yes | Unclear | No | Yes | NA | Yes | Moderate |
| Elabbasy et al., 2024 | Prospective cross-sectional cohort study | JBI Analytical Cross-Sectional Checklist | Yes | Yes | Unclear | Yes | Yes | Yes | Yes | NA | Yes | low |
| Albudayri et al., 2024 | Retrospective cross-sectional study | JBI Analytical Cross-Sectional Checklist | Yes | Yes | Unclear | Unclear | Unclear | No | Yes | NA | Yes | Moderate |
| AlSaleh et al., 2023 | Retrospective surveillance study | JBI Prevalence Checklist | Yes | Yes | Yes | Yes | NA | NA | Yes | NA | No | Low |
| Alwazzeh et al., 2023 | Retrospective observational study | JBI Cohort Checklist | Yes | Yes | Yes | Yes | Yes | No | Yes | Unclear | Yes | Low |
| Almazeedi et al., 2023 | Retrospective cross-sectional study | JBI Analytical Cross-Sectional Checklist | Yes | Yes | Yes | Yes | No | No | Yes | NA | No | Moderate |
| Al-Sofyani and Uddin, 2022 | Retrospective cohort study | JBI Cohort Checklist | Yes | Yes | Yes | Yes | Yes | Yes | Yes | Yes | Yes | Low |
| Mazi et al., 2021 | Prospective cohort study | JBI Cohort Checklist | Unclear | Yes | Yes | Yes | Unclear | No | Yes | Yes | Yes | Moderate |
| Al-Tawfiq et al., 2023 | Retrospective surveillance study | JBI Cohort Checklist | Unclear | Unclear | Yes | Yes | No | No | Yes | Unclear | Yes | Moderate |
| Alshamrani et al., 2024 | Retrospective surveillance study | JBI Cohort Checklist | Yes | Yes | Yes | Yes | Unclear | No | Yes | Unclear | Yes | Moderate |
| Saleem et al., 2023 | Prospective cohort study | JBI Cohort Checklist | Unclear | Yes | Yes | Unclear | No | No | Yes | Unclear | No | High |

*Abbreviations: JBI, Joanna Briggs Institute; NA, not applicable.*

*Note: Different JBI critical appraisal checklists were applied according to study design; therefore, some domains were not applicable to certain studies.*
